# Methodological Concerns Regarding Levels of Vascular Endothelial Growth Factor (VEGF) in Serum of Women with Endometriosis

**DOI:** 10.22074/ijfs.2015.4188

**Published:** 2015-02-07

**Authors:** Umberto Leone Roberti Maggiore, Simone Ferrero

**Affiliations:** Department of Obstetrics and Gynecology, San Martino Hospital and National Institute for Cancer Research, University of Genoa, Genoa, Italy

We read with interest the manuscript entitled "Serum and peritoneal fluid levels of vascular endothelial growth factor in women with endometriosis" recently published in the International Journal of Fertility and Sterility (1). The authors measured the levels of vascular endothelial growth factor in serum and peritoneal fluid of 179 women, 90 of whom were diagnosed with endometriosis, and observed that serum VEGF levels were similar in both study groups while women with endometriosis had higher levels of VEGF in peritoneal fluid compared with controls. Thus, the authors have concluded that endometriosis "is not related to the change of VEGF level in circulation". We believe that the methodology used in the study was not adequate to establish levels of "circulating" VEGF at the time of sampling thus invalidating the results of the study.

It is well known that VEGF is stored in the alpha granules of circulating resting platelets and released during clotting. Therefore, platelet activation and degranulation *in vitro* is the main contributor to the level of serum VEGF and may not reflect the level of circulating VEGF produced by peripheral tissues (such as endometriotic lesions) (2). In the study by Kianpour et al. (1), small differences in the circulating VEGF between women with and without endometriosis may have been masked by the large quantity of VEGF released by platelets during clotting. Based on these observations, plasma (preferably citrate, theophylline, adenosine and dipyridamole, CTDA plasma) should be preferred to serum for the assessment of circulating extracellular VEGF (3).

Furthermore, when serum is used for the measurement of VEGF, the methodology of collection and processing of samples should be standardised and declared. In fact, spinning the samples for different times and with different forces may influence the levels of VEGF (4). Furthermore, the interval between sample collection and processing (clotting duration) influences the levels of VEGF in supernatant, in particular when samples are processed after more than two hours from collection (4).

Given these considerations, we believe that the authors’ conclusion that "circulating" VEGF is similar in blood of women with and without endometriosis is not supported by the presented data.
